# Genomic analysis of three medieval parchments from German monasteries

**DOI:** 10.1038/s41598-025-86887-y

**Published:** 2025-01-25

**Authors:** Felix Heinrich, Henner Simianer, Jörg Bölling, Hedwig Röckelein, Christian Roos, Christian Reimer, Armin O. Schmitt

**Affiliations:** 1 Breeding Informatics Group, Department of Animal Sciences, Georg-August University, 37075 Göttingen, Germany; 2 Center for Integrated Breeding Research (CiBreed), Georg-August University, 37075 Göttingen, Germany; 3 Seminar für Mittlere und Neuere Geschichte, Kulturwissenschaftliches Zentrum, Georg-August University, 37073 Göttingen, Germany; 4 Institut für Katholische Theologie, Universität Hildesheim, 31141 Hildesheim, Germany; 5 Gene Bank of Primates and Primate Genetics Laboratory, German Primate Center, Leibniz Institute for Primate Research, 37077 Göttingen, Germany; 6 Animal Breeding and Genetics Group, Department of Animal Sciences, Georg-August University, 37075 Göttingen, Germany; 7 Institute of Farm Animal Genetics, Friedrich-Loeffler-Institut, 31535 Neustadt, Germany

**Keywords:** Genetics, Genomics

## Abstract

In the last two decades there has been growing interest in the analysis of ancient DNA obtained from the parchment used in historic documents. The genetic insight that this data provides makes collections of historic documents an invaluable source for studying the development and spread of historical livestock populations. Additionally, the biological data may provide new information for the historical analysis that could be used to determine the provenance as well as the authenticity of these documents. In this study, we extracted DNA from three medieval parchments that were written in German monasteries in the twelfth century. The source animal of the parchments could be identified as cattle and we compared their genome sequences with those of modern populations that are part of the 1000 Bull Genomes Project. The three animals were found to carry mtDNA haplogroup T3 and show a closer genetic relationship to other historic animals than to modern breeds. We further identified 39 haplotypes and 132 SNPs variants, which are rare ($<0.1$) or even non-existent in modern breeds. Finally, the genetic distances between the parchment samples show a putative association with the dates when the documents were written, indicating the usefulness of genetic analysis for provenance research.

## Introduction

The parchment of historic documents such as medieval charters or even older manuscripts like the Dead Sea scrolls forms a resource of biological information that allows us to gain insight into the genome of the animals whose hides were used^1,2^. Parchment, typically made from the hides of domestic animals like cattle, sheep, and goats, is crafted in a process where the hides are stretched, dried, and scraped to produce a durable writing surface that can last for centuries^3,4^. In the last two decades, researchers have started to extract genetic material in the form of DNA from this material to study the genomes of these animals^5–7^. While the first DNA extraction protocols were destructive and therefore limited in their usage, in recent years new less destructive and invasive methods have been developed such as rubbing the parchment surface with a PVC eraser^3,8–10^. Compared to other sources of historical DNA such as bones or teeth, parchment proved to be superior both in terms of the amount of endogenous DNA obtained and the accuracy of the dating information^1^. The genetic data extracted from parchment may reveal information regarding the geographical and temporal genetic variation of historical livestock populations and their development as well as the characteristics of historic animals^1,11^. Beside this biological perspective, this information is also valuable for historians. Traditionally, the source species for parchments are identified based on the hair grain patterns. However, for many parchments it might not be possible to identify the source species using this methodology^12^. Genetic information can help to identify the source species and may additionally shed light on the provenance of the animals. This would be helpful for the detection of forgeries as well as for dating and comparing documents found in the same location^11^. For example, in their study of the Dead Sea scrolls, Anava et al. analyzed the relationships between the different fragments and scrolls based on the genetic information extracted from the material^2^.

In this study we analyzed the parchment of three medieval charters (DA28, DA40 and DA45) from the twelfth century that are part of the main charter collection of the Diplomatic Apparatus of the Georg-August University Göttingen^13^. DA28 is one of three versions of a document allegedly issued by King Lothar III to the Provost of the Augustinian Canons of Riechenberg near Goslar (Lower-Saxony) and his bailiff. It concerns the protection and confirmation of church ownership for several properties. The three versions of the charter slightly differ in their content^14^. DA40 has been allegedly issued by the Bishop Henry of Paderborn (North Rhine-Westphalia) towards the local Benedictine Abbey Abdinghof and confirms several donations of the former^15^. The last document, DA45, has been issued by the Bishop Bernhard of Hildesheim (Lower-Saxony) to the same Augustinian Canons of Riechenberg as document DA28. In it the jurisdiction and rights of a newly built church in Hanenthorp near Goslar are clarified^16^. All three documents are recipient copies, with DA28 and DA40 being a forgery resp. a suspected forgery while DA45 is an authentic copy. For more information about these charters see Supplementary Note.

To determine the age and provenance of animal hides used for historical documents, it is crucial to ascertain whether they are authentic documents or forgeries and to identify in which chancery they were written. Both questions can be more or less clearly answered using the hermeneutic methods of diplomatics and critical analysis of charters. According to medieval customs, authentic documents could be issued in either the issuer’s or the recipient’s chancery. DA45 is an authentic document but was not drafted in the chancery of the issuer, the Bishop of Hildesheim, but rather in the chancery of the recipient, the Augustinian Canon’s Abbey of Riechenberg near Goslar (Lower-Saxony)^17^. Forged documents, however, such as DA28 and presumably also DA40, consistently originate from the chancery of the recipient; in the first-mentioned case, from Riechenberg, and in the second case, from the Benedictine Abbey Abdinghof in Paderborn (North Rhine-Westphalia). Depending on the situation, the issuer or the recipient procured the hides from the regional market or their own estates.

The hides for DA28 and for DA45, both of which are recipient’s copies drafted in the abbey of Riechenberg, are likely to have come from cattle in the Harz Mountains or its foothills. For DA40 it is irrelevant for the provenance of the hides whether it is a genuine or a forged document, as both the Bishop of Paderborn and the Abbot of Abdinghof are likely to have obtained their parchment from the same region.

For dating the parchments, distinguishing between authentic and forged documents is also significant. In the case of an authentic document, the age of the parchment corresponds to the document’s issuance date; for DA45, this would be November 5, 1133. For forged documents, however, there is typically a gap between the stated and the actual issuance dates. The document DA28 claims to have been issued on February 7, 1131. There are three versions of the document, which were all forged at the Abbey of Riechenberg. The version that was tested in our study has most likely been forged at the end of the 1180s^18^. The document DA40 purports to have been issued on March 26, 1103, by Bishop Henry of Paderborn. This forgery was likely made on the order of, or even personally by, Abbot Konrad of Abdinghof, who held this position from 1142 to 1173^19^.

To obtain insight into the source animals of the three documents, we extracted and sequenced DNA taken from small samples of the parchments. After identifying the species as cattle we compared their genetics to other ancient animals^20–22^ and animals of the 1000 Bull Genomes Project^23^, a collection of whole-genome sequencing data from more than 6000 individuals that cover a significant proportion of the modern cattle breeds. We built a phylogenetic tree comprising the three parchment animals and the breeds presented in the 1000 Bull Genomes Project. Furthermore, we identified alleles and haplotypes that are no longer common among modern animals. Lastly, we interpreted the genetic similarity of the parchment animals in context of the provenance of the documents.

## Materials and methods

### Sample extraction and sequencing

A small piece of parchment was taken from the corners of three medieval charters written on animal hides on November 26, 2019. Two of these charters (DA28 and DA45) originate from the Augustinian Canon’s Abbey of Riechenberg in Goslar, Lower-Saxony, Germany and the third one (DA40) originates from the Benedictine Abbey Abdinghof in Paderborn, North Rhine-Westphalia, Germany. DNA extraction and library preparation were performed in the ancient DNA laboratories of the German Primate Center in which all standards for such laboratories were implemented (e.g., UV light decontamination before and after use, positive air pressure, separate sterile working areas, protective clothing, negative controls during DNA extraction and sequencing library preparation). For DNA extraction, we applied a column-based method specifically designed to recover degraded DNA fragments^24,25^. After extraction, DNA concentrations were measured with a Qubit 4.0 fluorometer (ThermoFisher Scientific, USA), and DNA quality and degradations were checked on a Bioanalyzer 2 100 (Agilent Technologies, USA). Genomic DNA (50 ng) was then used to construct shotgun sequencing libraries with the NEBNext Ultra II DNA Library Prep Kit (New England Biolabs, USA). All standard protocols of the manufacturer were followed, except DNA fragmentation before library preparation was omitted due to the degraded status of the DNA. After end repair, adapter ligation, and ligation cleanup without size selection, libraries were indexed with multiplex oligos and then cleaned with kit’s purification beads. Library concentration and size distribution were measured with a Qubit fluorometer and Bioanalyzer, respectively, and molarity was quantified via quantitative polymerase chain reaction (qPCR) using the NEBNext Library Quant Kit (New England Biolabs, USA). Sequencing was conducted on an Illumina HiSeq 4000 (50 bp single-end read) at the NGSIntegrative Genomics Core Unit (NIG) of the University Medical Center Göttingen (Germany). Raw sequencing reads were demultiplexed with Illumina software. Sequencing adapter remnants and low quality reads were removed using AdapterRemoval version 2.3.1^26^.

### Animal species identification of parchments

In order to identify the species of the samples and the amount of contamination in the DNA we used FastQ Screen v0.15.3^27^. Using bowtie2 version 2.5.1^28^ as aligner this tool aligns the sequenced reads with the following reference genomes: cattle (Bos_taurus.ARS-UCD1.2), goat (Capra_hircus.ARS1), pig (Sus_scrofa.Sscrofa11), sheep (Ovis_aries.Oar_v3.1.75), and human (Homo_sapiens.GRCh38). The reference genomes were obtained from the Ensembl database^29^. The amount of bacterial contamination was accounted for by using a representative database of 112 bacterial genomes, which was downloaded from http://progenomes.embl.de/representatives.cgi^30^ on June 12, 2020.

### Assembly of mitochondrial genomes

After demultiplexing the raw sequencing reads with Illumina software, the following bioinformatics analysis was performed with the Geneious Prime 2023.0.1 package (https://www.geneious.com/). First, we trimmed and quality-filtered reads with BBDuk v38.84 of the BBTools package (https://jgi.doe.gov/data-and-tools/bbtools/) and then removed duplicate reads with Dedupe v38.84 (BBTools package), both with default settings. We then mapped reads onto the *Bos taurus* mitochondrial reference genome (GenBank accession No. NC_006853) using the Geneious Assembler with default settings (maximum five iterations). The newly produced mitochondrial genomes were manually checked and annotated with Geneious. To identify bovine haplogroups and to trace the most similar mitochondrial genomes in GenBank we used the Basic Local Alignment Search Tool (BLAST; https://blast.ncbi.nlm.nih.gov/Blast.cgi)^31^.

### Ancient cattle genomes

For further comparison, we included published sequencing data from ancient cattle originating from various regions and time periods in our analysis. This dataset comprises 66 animals sequenced by Verdugo et al.^20^, which were sampled in Near Eastern countries; two Western European animals published by Erven et al.^21^ and 24 Iberian cattle provided by Günther et al.^22^. Among these, 14 animals were identified as belonging to *Bos primigenius*, while the remainder belong to *Bos taurus*. A detailed overview of the animals, including the sampling sites and their estimated ages, is provided in Supplementary Table S1.

### Variant calling

For variant calling we have combined our parchment samples with the sequences of 92 published ancient cattle samples. The filtered sequence reads were aligned to the *Bos taurus* reference genome version ARS-UCD1.2^32^ using bowtie2 version 2.5.1^28^ with the settings--end-to-end and--sensitive as recommended by Poullet et al. for mapping ancient DNA^33^. The alignment files were then sorted with samtools 1.17^34^ and duplicates were removed using the Picard toolkit 3.0.0^35^. To account for possible damages to the ancient DNA, we applied mapDamage 2.2.1^36^ to rescale the base quality scores according to their probability of being damaged. Sequencing coverage and depth at each position was determined using samtools. The 95 samples were then merged and variant calling was performed using bcftools 1.17^37^. We excluded insertions and deletions as well as non-biallelic single nucleotide polymorphisms (SNPs). Due to large differences in the genome coverage for the ancient samples, we removed samples with a low coverage of the genome from further analysis. As a final filtering step we restricted the SNPs to those located in high coverage regions on the autosomal chromosomes, i.e., those positions that have a read depth of at least 10 for each of the three parchment samples and the high coverage ancient samples.

### 1000 bull genomes project

Run 9 of the 1000 Bull Genomes Project^23^ consists of more than 150 million SNPs for 6191 animals that are assigned to 261 different breeds. Table 1 shows the ten most frequent breeds in the dataset, while a comprehensive list of animals, along with their respective breeds and species, is provided in Supplementary Table S2. Of particular interest are two breeds labeled as *Ancient* and *Auroch*. The sole animal assigned to the *Auroch* breed (SampleID: SAMN04028906) is a British auroch estimated to be approximately 6759 years old, sequenced from a bone sample^38^. The *Ancient* breed consists of two animals: Win1 (SampleID: UNKNLDF000000000025), an early medieval cow from the Netherlands, also published by Erven et al.^21^, and Bri1 (SampleID: UNKNLDM000000000036), an unpublished bull from the Roman period in the Netherlands. As Win1 is part of the ancient animals that we used for variant calling, we excluded it from the 1000 Bull Genomes Project dataset.

**Table 1:** Ten most frequent breeds in Run 9 of the 1000 Bull Genomes Project dataset.

Breed	#Individuals
Holstein	1148
Angus	401
NorwegianRed	347
BrownSwiss	294
Brahman	225
Jersey	195
Fleckvieh	162
Charolais	154
Hereford	142
Simmental	137

As a first step, we applied Beagle 5.4^39^ with an effective population size *ne* of 1000 as recommended by Pook et al.^40^ on each chromosome to impute missing values. The SNPs were then filtered to the high coverage regions identified for the parchment and ancient samples. Finally, the Run 9 dataset was merged with the parchment and ancient samples using PLINK v1.9^41^. Missing genotypes were assumed to be homozygous for the reference allele.

### Phylogenetic analysis

To study the relationship between the parchment samples, the ancient cattle and the breeds available in the 1000 Bull Genomes Project a phylogenetic tree was built using the merged dataset from the previous section. For each breed we created a representative. The genotypes of this individual were defined as the mode of all individuals that belong to the specific breed. 360 animals with breed labelled “unknown” were excluded from this analysis. PLINK v1.9 was employed to calculate distances between the breed representatives and the parchment samples defined as $1 - \text {identity-by-state}$ (--distance 1-ibs). The R packages ape 5.7.1^42^ and ggtree 3.10.0^43^ were then applied to perform neighbor-joining^44^ on the distance matrix and to visualize the resulting phylogenetic tree. To verify the consistency of the trees’ edges and nodes, we ran 100 bootstrap replicates using the function boot.phylo from ape. Additionally, we performed a principal component analysis (PCA) based on the more frequent taurine breeds in the 1000 Bull dataset, which occur with at least 40 individuals. A PCA plot was created and the parchment and ancient samples were then projected on this plot. PLINK v1.9 was used to perform the PCA only on the modern breeds with the option--pca-clusters. Lastly, we calculated outgroup $f_{3}$-statistics using the ADMIXTOOLS 2 2.0.8 R package^45^ and the British auroch as outgroup. For each of the three parchment samples, we estimated $f_{3}$-statistics of the form $f_{3}(\text {British auroch: Parchment sample, X})$, where X represents all the individuals of 1000 Bull Genomes Project except for Bri1.

### Rare alleles and haplotypes

To identify haplotypes the merged dataset was initially phased using Beagle 5.4^39^. Haplotype blocks were then identified using the R package LDExplorer^46^ with the blocks being defined based on the D’ coefficient of linkage disequilibrium as proposed by Gabriel et al.^47^. We then defined those alleles and haplotypes as rare that occur in at least one of the three parchment samples heterozygously and which have a frequency of less than 10% in the merged dataset. Due to the haplotype blocks not covering all SNPs we applied the same approach on the remaining single SNPs to identify alleles that occur in the parchment samples but are rare in the merged dataset.

## Results and discussion

### DNA extraction and sequencing

By sequencing the parchment samples using an Illumina HiSeq 4000 platform, we obtained approximately 65, 184, and 113 million sequence reads for the samples DA28, DA40, and DA45, respectively. The Illumina sequencing platforms have previously been successfully used for sequencing ancient DNA^1,3,48–50^. By clipping adapters and removing low quality reads using AdapterRemoval between 2600 and 9800 reads were removed from these raw reads for each sample.

### Identification of parchment source

**Fig. 1:**
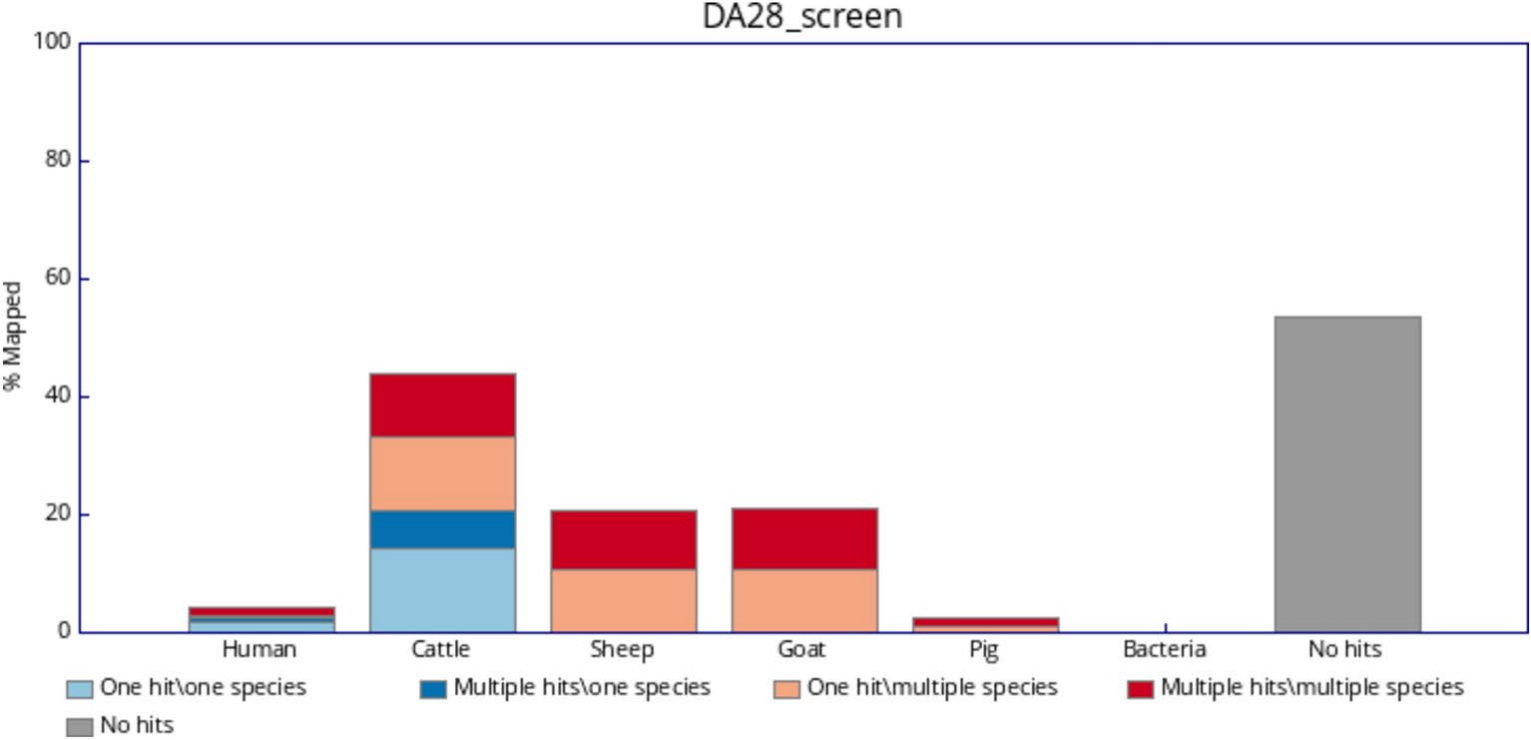
Percentage of reads of parchment DA28 (i) aligned uniquely to one genome only (light blue), (ii) aligned multiple times to one genome only (dark blue), (iii) aligned uniquely to a given genome and at least one other genome (light red) or (iv) aligned multiple times to a given genome and at least one other genome (dark red).

**Fig. 2:**
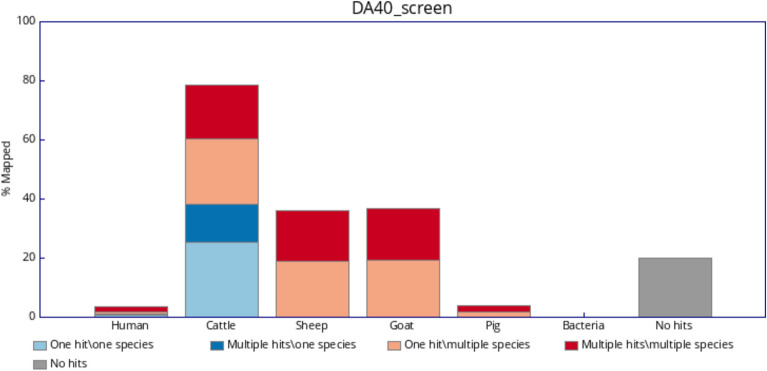
Percentage of reads of parchment DA40 (i) aligned uniquely to one genome only (light blue), (ii) aligned multiple times to one genome only (dark blue), (iii) aligned uniquely to a given genome and at least one other genome (light red) or (iv) aligned multiple times to a given genome and at least one other genome (dark red).

**Fig. 3:**
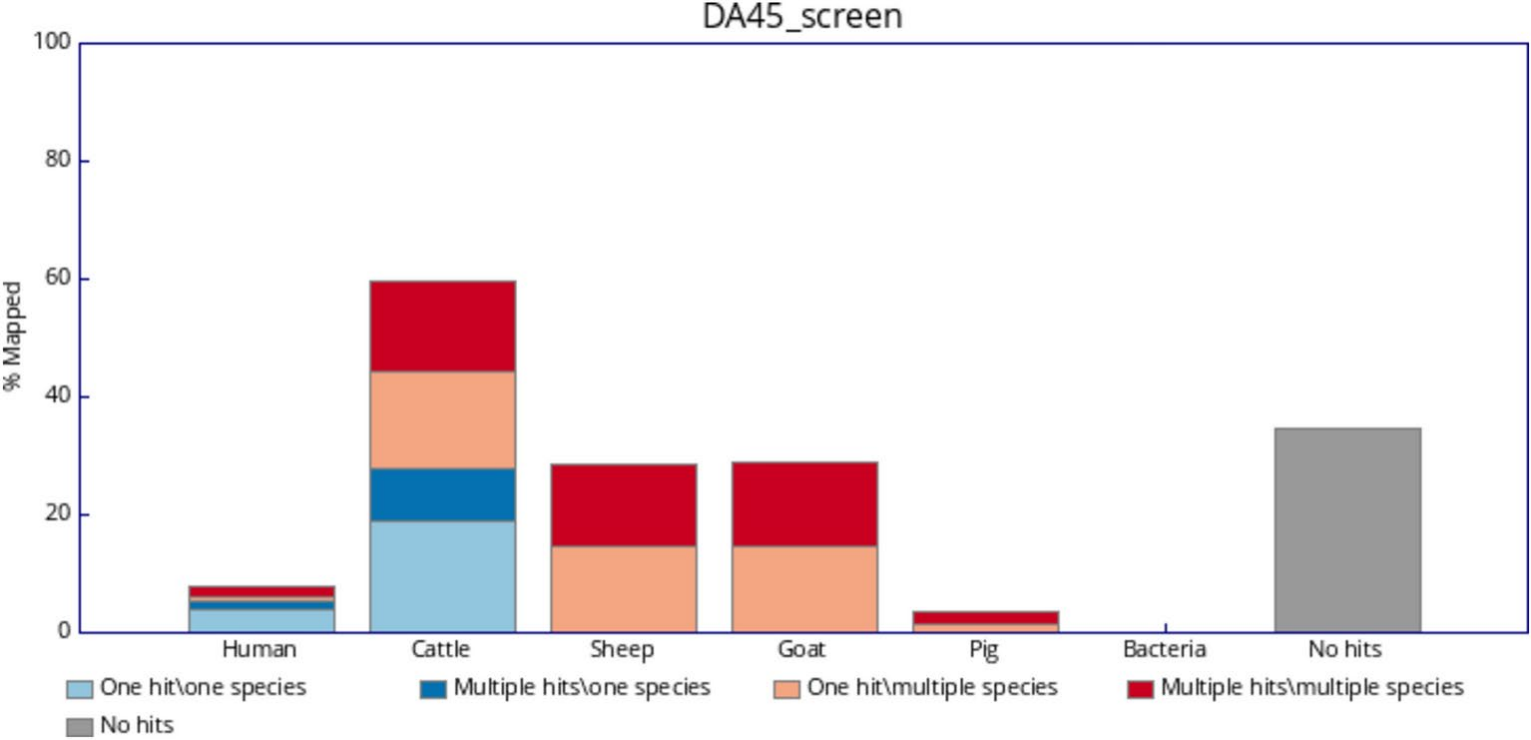
Percentage of reads of parchment DA45 (i) aligned uniquely to one genome only (light blue), (ii) aligned multiple times to one genome only (dark blue), (iii) aligned uniquely to a given genome and at least one other genome (light red) or (iv) aligned multiple times to a given genome and at least one other genome (dark red).

In order to identify the animal species of the hide used for creating the investigated parchments, we applied FastQ Screen^27^ to align the sequence reads with the reference genomes of the animal species most commonly used in the manufacture of parchments, namely cattle, goat, pig and sheep. Additionally the reads were aligned with the human genome as well as with a representative set of bacterial genomes to account for possible contamination of the samples. The results for the three parchments can be seen in Figs. 1, 2 and 3. For all three samples the majority of reads that align only with a single genome (light and dark blue, with dark blue indicating repetitive DNA sequences) are aligned with the cattle reference genome marking it as the most likely species of origin for the parchments. These comprise between 20.89 and 38.33% of the reads. A minor amount of reads (0.91–5.3%) map only to the human genome. This contamination of the samples may be explained due to human handling of the documents. Teasdale et al. for example observed an average of 5.6% of human DNA in legal documents and an even higher average of 11.1% in more frequently handled religious manuscripts^9^. Between 22.97 and 40.36% of the reads align to the cattle genome as well as to the genomes for sheep and goat (light and dark red). This cross alignment may be explained due to the high degree of sequence similarity, in particular among repeated elements, between the genomes of these ruminants and has also been observed in an analysis of parchments from the seventeenth and eighteenth century^1^. The amount of bacterial contamination observed is basically zero for all three samples.

### Mitochondrial genomes

We generated complete mitochondrial genomes from all three samples with high sequencing depth (DA28: 16,340 bp, 73x; DA40: 16,341 bp, 145x; DA45: 16,341 bp, 85x). Mitochondrial genomes differed from each other in 5-11 positions (DA28-DA40: 9 positions; DA28-DA45: 5 positions; DA40-DA45: 11 positions). BLAST search revealed that all three mitochondrial genomes belong to haplogroup T3^51^ with 99.95–99.98% identity to the most similar sequence in GenBank (DA28: 99.98% identity to MT576766, 3 positions different; DA40: 99.96% identity to MZ901520, 6 positions different; DA45: 99.95% identity to MN200836, 8 positions different).

### Variant calling

Having identified the animal origin of the parchments as cattle we aligned the reads with the cattle reference genome using bowtie2^28^. The bowtie2 aligner was found to be well appropriate for ancient DNA^33^. The overall alignment rates were 44, 79, and 59% for the samples DA28, DA40, and DA45, respectively, which corresponds to the results shown in Figs. 1, 2 and 3. After the removal of duplicates using the Picard toolkit, we were left with 26.4, 124.2, and 60.0 million reads for the samples DA28, DA40, and DA45, respectively. The average coverages after filtering the reads were 0.42x, 2.06x and 0.97x across the autosomes and the covered fractions of the genome were 33.6, 84.4, and 59.7% for DA28, DA40, and DA45, respectively, which are markedly higher than the mean coverage of 0.01x for $\sim$ 2000 years old dead sea scrolls made mostly from sheep skin, which were recently analyzed^2^. All three parchment samples come from male animals with the mean coverage for the sex chromosomes being roughly half the coverage of the autosomal chromosomes. The 92 ancient cattle samples were aligned and prepared using the same methods. The average coverages across autosomes and covered fractions of the genome for these samples are detailed in Supplementary Table S1. Notably, due to significant variation in the number of sequencing reads per sample, the covered fraction of the genome differs substantially between the individuals, ranging from less than 1% to more than 99%. Variant calling with bcftools^37^ yielded an initial set of 16,931,459 biallelic SNPs. We excluded ancient cattle samples if their covered fraction of the genome was less than 75%, which left us with 16 samples. As a final filtering step we restricted our analysis to the regions with high coverage (read depth $\ge 10$) on the autosomal chromosomes across the 16 ancient samples and three parchment samples. This resulted in 803 high-quality SNP markers for the combined samples. Additionally, we created a second dataset without the restriction to high coverage regions where we removed all SNPs with missing genotypes for the 16 ancient and three parchment individuals. This second dataset consists of 828,543 SNPs.

### 1000 bull genomes project

In order to perform phylogenetic analysis of the parchment and ancient samples and to study the relationship between these old animals and modern breeds we merged the region restricted dataset with the data from Run 9 of the 1000 Bull Genomes Project^23^. The 1000 Bull Genomes Project data contains 21,138 SNPs that are located in the high coverage regions we identified for the parchment and ancient samples. Of these, 606 SNPs overlap with the 803 high-quality SNPs found for the samples. Merging the two datasets resulted in a final dataset of 21,329 SNPs for 6209 animals. Supplementary Table S3 lists the number of SNPs for each chromosome in this dataset.

### Phylogenetic analysis

**Fig. 4:**
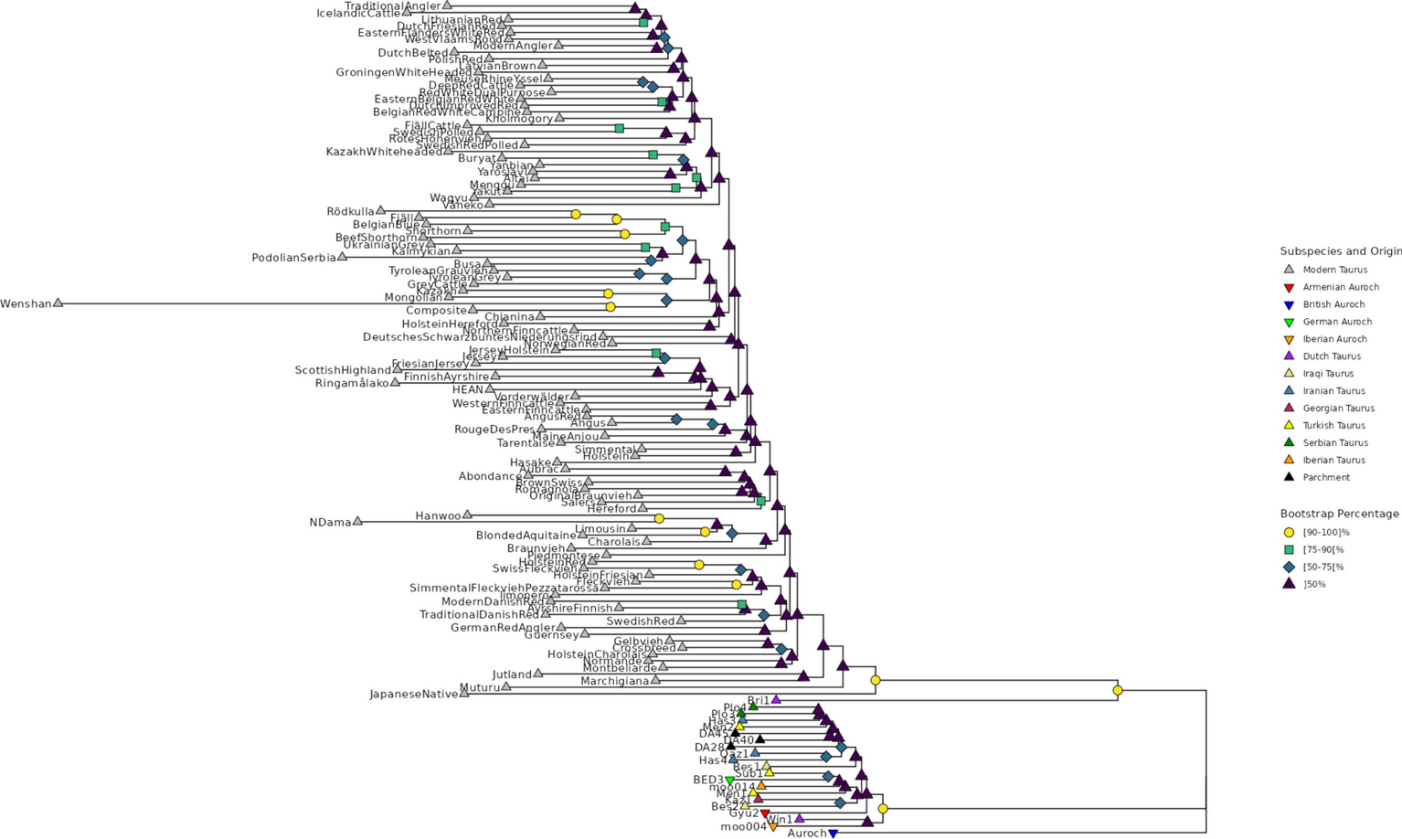
Phylogenetic tree of the taurine cattle breeds present in Run 9 of the 1000 Bull Genomes Project, the 16 ancient cattle samples and the three parchment samples. Each modern breed is represented by an individual based on the mode of the genotypes of all individuals belonging to that breed. The color and shape of the tip points indicate the subspecies and geographic origin of the breed/individual while the color and shape of the nodes show the proportion of 100 bootstrap replicates that reproduced each branch point. The British auroch sample has been used as root.

To visualize the relationship between the different breeds as well as the ancient and parchment samples we created a phylogenetic tree based on a single representative for each breed as well as the individual ancient and parchment samples. This tree is shown in Fig. 4 with the tip points colored and shaped according to the subspecies and geographic origin while the color and shape of the nodes show the proportion of bootstrap replicates that reproduced them.

**Fig. 5:**
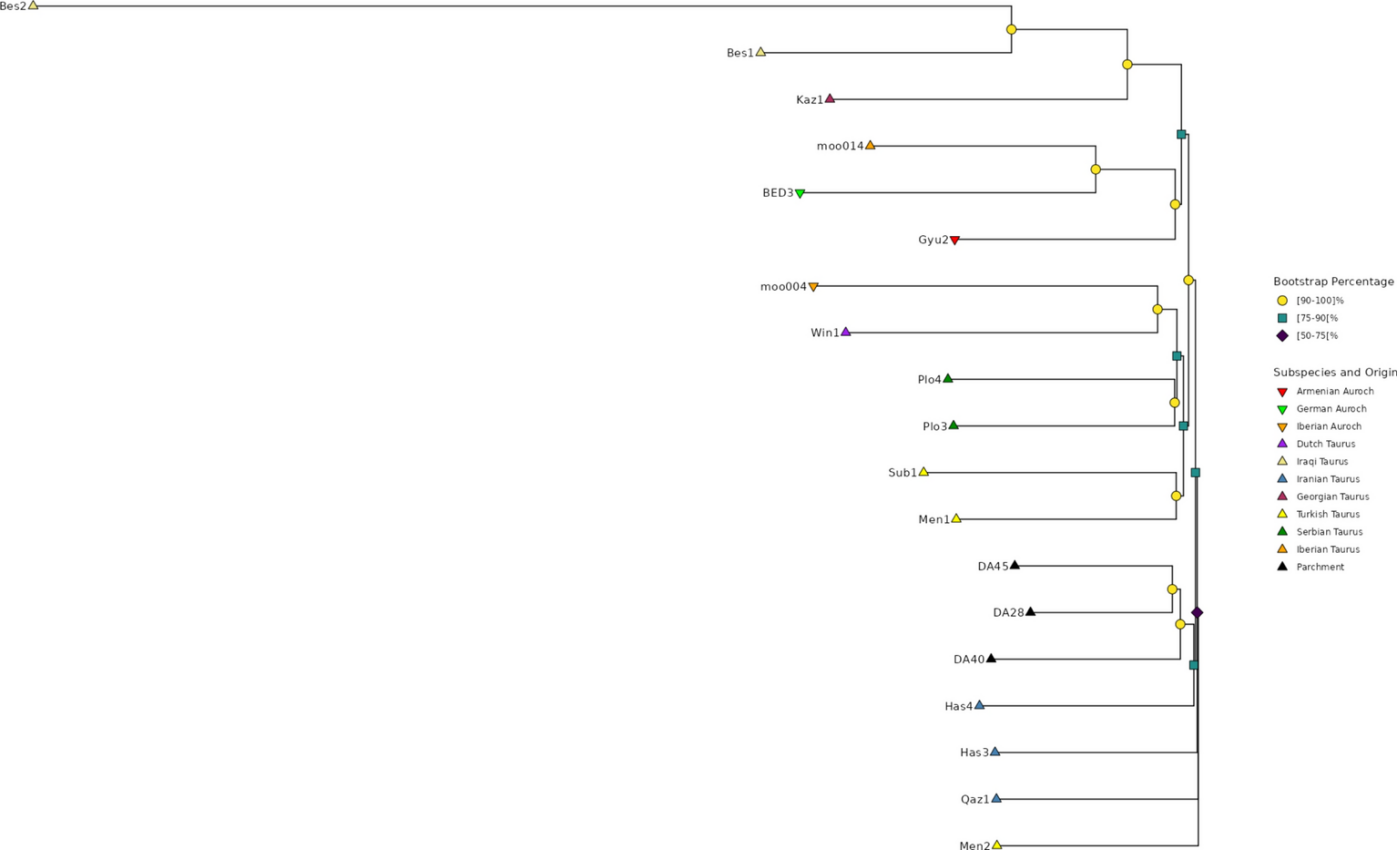
Phylogenetic tree of the 16 ancient cattle samples and the three parchment samples based on 828,543 SNPs. The color and shape of the tip points indicate the subspecies and geographic origin of the individual while the color and shape of the nodes show the proportion of 100 bootstrap replicates that reproduced each branch point.

Due to the large number of breeds—many of which only occur with one (54) or two (28) animals in the 1000 Bull Genomes Project dataset—we restricted the tree to those taurine breeds which have at least five individuals available. Additionally, we used from the 1000 Bull dataset the single 6750-year-old British *Auroch* sample, which was used as root for the tree, and the Bri1 sample, which is the single remaining *Ancient* sample and originates from the Roman period in the Netherlands. The parchment and ancient samples form a distinct group on a branch that is separated from the modern cattle breeds with Bri1 being between these two parts of the tree. However, the distances between the parchment and ancient samples in this tree are derived from the relatively small number of 803 SNPs as these individuals are assumed to be homozygous for the reference allele for the SNPs from the 1000 Bull Genomes Project dataset. Consequently, the genetic distances may be less reliable. To address this limitation and provide a better visualization, we constructed a second phylogenetic tree with only the ancient and parchment animals based on our second dataset with 828,543 SNPs. This tree, depicted in Fig. 5, places the three parchment samples on a distinct branch, with DA28 and DA45 showing greater genetic similarity to each other than to DA40. The next closest individuals are four ancient *Bos taurus* samples from Iran and Turkey.

To further explore the genetic relationships between ancient and modern animals, we performed a PCA based on the modern taurine cattle, projecting the ancient and parchment samples onto the resulting principal components. To visually distinguish the modern breeds, we included only breeds represented by at least 40 animals in the PCA. The resulting plot is presented in Supplementary Fig. S1. The parchment samples and most of the ancient samples are located very close together, with no clear separation between *Bos taurus* and *Bos primigenius* individuals. This limitation of a PCA based solely on modern genetic variation, which might not fully capture the genetic variation among the ancient animals, has previously been noted by Günther et al.^22^. Moreover, the PCA has limited explanatory power, as the first two principle components account for only 1.69% of the total genetic variation. Following the approach of Ciucani et al.^52^, we calculated outgroup $f_{3}$-statistics to assess shared genetic drift between the three parchment samples and modern individuals of the 1000 Bull Genomes Project, using the British auroch as the outgroup. The 20 modern individuals with the highest $f_{3}$-statistics for each parchment sample are listed in Supplementary Table S4. There is considerable overlap among the closest individuals for all three parchment samples, with the top five belonging to various Northern European breeds, such as Holstein, Ayrshire Finnish, Swedish Red, and Angus. However, these are followed by a mix of different taurine but also indicine individuals. In summary, it is not possible to confidently link the parchment animals to a specific modern breed. This ambiguity may stem from the relatively small number of SNPs used to compare ancient and modern animals.

### Rare alleles and haplotypes

We were interested to find variants that are present in the parchment samples but which are rare among the modern cattle breeds. Such variants may have become less frequent or even lost due to the effects of selective breeding or genetic drift. After phasing of the dataset using Beagle^39^ and determining haplotype blocks using LDExplorer^46^ we obtained 1197 blocks that cover in total 3589 out of the 21,329 SNPs. For these blocks there are 39 haplotypes that occur in at least one of the parchment samples while being rare ($<0.1$) among the animals collected by the 1000 Bull Genomes Project. A list of the corresponding blocks with their respective haplotypes for the parchment samples can be found in Table 2. The majority of the SNPs are not covered by the identified haplotype blocks. For this reason, we also analyzed the individual SNPs and identified those with alleles in the parchment samples that occur only rarely ($<0.1$) among the modern animals. In total, there are 132 SNPs that have such rare alleles in the parchment samples. These SNPs and their corresponding genotypes for the parchment samples can be found in Supplementary Table S5. We have further checked for the polledness of the three animals, in particular whether they have the *Celtic* or *Friesian* mutations^53–55^. These mutations consist of deletion and duplication events in specific regions. Based on the read depth of these regions, which is comparable to the remainder of the autosomal chromosomes, we conclude that these animals did not have these sequence variants. Therefore, it is most likely that these animals were horned.

**Table 2:** Haplotype blocks where at least one of the three parchment samples have a rare ($<0.1$) haplotype. For each block, the haplotypes of the parchment samples are given with their respective frequency among all individuals in the dataset.

Chr	Start (bp)	End (b)	#SNPs	DA28	DA40	DA45
2	121,567,158	121,567,240	4	TGGG (0.900)/TGGG (0.900)	TGGG (0.900)/TGGG (0.900)	TGGG (0.900)/TGGA (0.080)
2	186,042	186,044	2	CG (0.011)/CG (0.011)	CG (0.011)/CG (0.011)	AG (0.000)/AG (0.000)
2	121,574,521	121,574,522	2	AG (0.001)/GG (0.002)	GG (0.002)/GG (0.002)	GG (0.002)/GG (0.002)
3	113,158,035	113,344,751	2	TT (0.059)/TT (0.059)	TT (0.059)/TT (0.059)	TT (0.059)/TT (0.059)
4	213,635	213,643	4	GGTC (0.072)/GGTC (0.072)	GGTC (0.072)/GGTC (0.072)	GGTC (0.072)/GGTC (0.072)
5	13,792,670	13,792,677	2	GA (0.982)/GA (0.982)	GA (0.982)/GT (0.002)	GA (0.982)/GA (0.982)
6	5,461,348	5,461,358	2	CG (0.092)/CG (0.092)	CG (0.092)/CG (0.092)	CG (0.092)/CG (0.092)
8	10,210,908	10,210,997	3	CCA (0.020)/CCC (0.490)	GCA (0.489)/CCC (0.490)	CCA (0.020)/CCC (0.490)
8	10,210,765	10,210,787	5	TAGTA (0.003)/TAGTA (0.003)	TAGTA (0.003)/TAGTA (0.003)	TAGTA (0.003)/TAGTA (0.003)
8	10,295,206	10,295,212	2	TT (0.071)/AT (0.503)	TG (0.426)/AT (0.503)	TT (0.071)/AT (0.503)
11	26,499,145	26,499,149	2	GT (0.004)/GT (0.004)	GT (0.004)/GT (0.004)	GT (0.004)/GT (0.004)
13	364,136	364,140	3	GCG (0.027)/GTA (0.501)	GTA (0.501)/GTA (0.501)	GTG (0.005)/GTA (0.501)
13	363,098	363,100	2	TG (0.499)/CT (0.499)	TT (0.001)/TG (0.499)	TG (0.499)/TG (0.499)
13	363,979	363,980	2	CA (0.936)/CG (0.059)	CA (0.936)/CA (0.936)	CA (0.936)/CA (0.936)
13	364,033	364,034	2	CG (0.496)/CT (0.128)	CG (0.496)/CT (0.128)	TG (0.004)/CT (0.128)
15	24,995,662	24,995,695	5	TCGCC (0.003)/TCGCC (0.003)	TCGCC (0.003)/TCGCC (0.003)	TCGCC (0.003)/TCGCC (0.003)
15	84,841,867	84,841,888	4	GAAG (0.002)/GAAG (0.002)	AAAG (0.997)/AAAG (0.997)	AAAG (0.997)/GAAG (0.002)
15	84,829,529	84,829,532	2	GA (0.003)/GA (0.003)	GA (0.003)/GA (0.003)	GA (0.003)/GA (0.003)
15	84,881,062	84,881,064	2	GG (0.023)/GG (0.023)	GG (0.023)/GG (0.023)	GG (0.023)/GG (0.023)
20	111,321	111,341	2	CA (0.046)/CA (0.046)	CA (0.046)/CA (0.046)	CA (0.046)/CA (0.046)
20	28,265,147	28,265,158	2	AA (0.038)/AT (0.536)	AT (0.536)/AA (0.038)	AA (0.038)/AT (0.536)
21	33,028,875	33,029,036	7	CGAGGAG (0.003)/CGAGGAG (0.003)	CGAGGAG (0.003)/CGAGGAG (0.003)	CGAGGAG (0.003)/CGAGGAG (0.003)
21	32,924,028	32,924,068	2	CC (0.504)/TC (0.005)	CC (0.504)/CC (0.504)	CC (0.504)/CC (0.504)
21	32,925,380	32,925,413	4	GTGG (0.002)/CTGG (0.498)	CTGG (0.498)/GTGG (0.002)	CTGG (0.498)/GTGG (0.002)
21	686,526	686,546	6	TCGGGA (0.001)/TCGAGG (0.003)	TCGAGA (0.498)/TCGGGG (0.048)	TCGAGA (0.498)/TCGAGG (0.003)
21	686,588	686,592	3	GTT (0.006)/ATC (0.034)	GTC (0.498)/GTC (0.498)	GTC (0.498)/GTC (0.498)
23	33,338,869	33,338,902	5	AGCGC (0.001)/AGCAC (0.586)	AGCGC (0.001)/AGCGC (0.001)	AGCAC (0.586)/AGCAC (0.586)
27	6,222,995	6,223,015	4	GGGG (0.037)/GGGC (0.605)	AGGC (0.334)/GGGC (0.605)	GGGC (0.605)/AGGC (0.334)
27	6,223,691	6,223,696	2	GC (0.001)/CA (0.999)	GC (0.001)/CA (0.999)	CA (0.999)/GA (0.000)
28	478,711	478,723	3	GTC (0.938)/GGC (0.000)	GTC (0.938)/GTC (0.938)	GTC (0.938)/GTC (0.938)
28	505,764	505,774	2	CG (0.050)/CG (0.050)	CG (0.050)/CG (0.050)	CG (0.050)/CG (0.050)
29	26,611,800	26,611,855	4	ATGC (0.003)/ATGC (0.003)	ATGC (0.003)/ATGC (0.003)	ATGC (0.003)/ATGC (0.003)

### Results in the context of provenance

Based on the provenance of the charters, as outlined in the introduction, one would expect that the hides of DA28 and DA45, which both originate in Riechenberg (Lower-Saxony), would be more similar to each other and that both would show greater distance from DA40, which originates in Paderborn (North Rhine-Westphalia). As the phylogenetic tree in Fig. 5 shows, the genetic distances reflect this assumption. The two documents DA28 and DA45 have the smallest genetic distance as their shared origin region would suggest. The distances are as follows:

- DA28 and DA40: distance of 0.0853450 (different genotypes at 130,083 SNPs)
- DA28 and DA45: distance of 0.0754216 (different genotypes at 118,068 SNPs)
- DA40 and DA45: distance of 0.089451 (different genotypes at 135,340 SNPs)

Additionally, the different distances to DA40 could be indicative of the temporal difference. DA45 is known to have been created in 1133, while DA28 was likely forged in the late 1180s. The suspected forgery DA40 is presumed to have been issued between 1142 and 1173, while it purports to have been issued in 1103. The greater genetic similarity between DA28 and DA40 than DA40 and DA45 might indicate that DA40’s issue date was closer to the late 1180s than 1133, which would validate the assumption of DA40 being a forgery. However, testing this idea would require further analysis and comparison with other contemporary parchments from these regions, preferably genuine documents with validated dates. While we have included several published ancient and historic cattle in our analysis these samples mostly originate from individuals that lived several thousand years earlier, limiting their direct relevance.

From both a diplomatic and biological viewpoint, comparing the documents investigated here with a genuine issuer’s document from a royal chancery and an issuer’s copy from the papal chancery of the twelfth century would also be a worthwhile prospect for future studies. Theoretically, one would expect an Italian provenance for the parchment of the papal document. Since the medieval king traveled regularly throughout his realm issuing documents, the parchment could have been sourced locally or from a larger supply that the chancery carried with them. Among the royal documents, those issued by Lothar III from his chancery in Goslar should be excluded, as they would likely again be hides from the Harz region. Examples from the chancery of the Salians or the Hohenstaufen would be better suited.

## Supplementary Information

Supplementary Information 1.

Supplementary Information 2.

Supplementary Information 3.

Supplementary Information 4.

Supplementary Information 5.

Supplementary Information 6.

Supplementary Information 7.

Supplementary Information 8.

## Data Availability

The sequence data of the three parchment samples is available from the European Nucleotide Archive (ENA) under the accession PRJEB76650.
