# Supplementary figures and images for: Genomic analysis of three medieval parchments from German monasteries
Source: Sci Rep. 2025 Jan 25;15:3156. doi: 10.1038/s41598-025-86887-y (PMC11759711; doi:10.1038/s41598-025-86887-y)

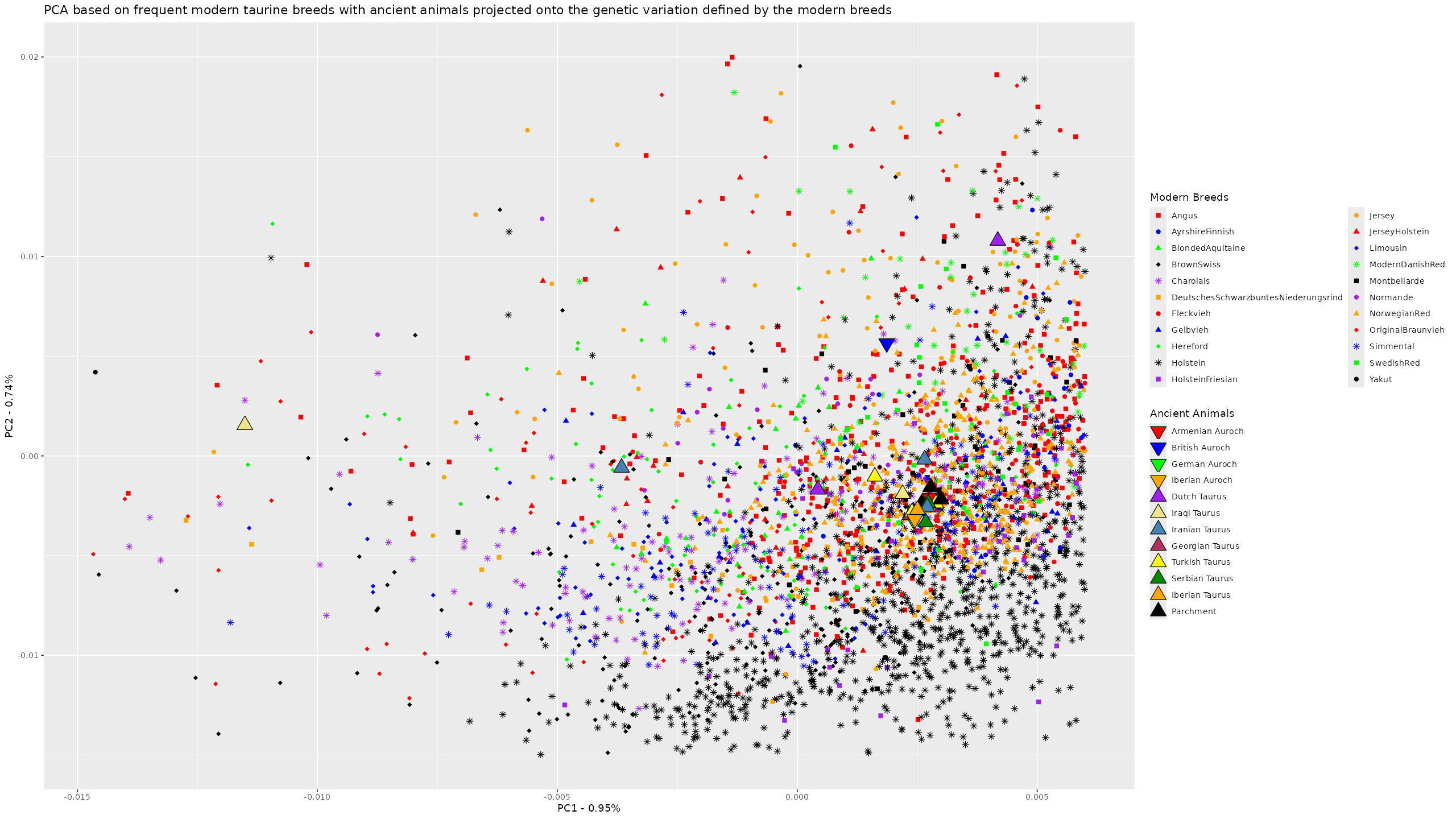

Supplement: Supplementary file 1 — Supplementary Information 1. [file 41598_2025_86887_MOESM1_ESM.png]
