# Supplementary material for: Genomic analysis of three medieval parchments from German monasteries
Source: Sci Rep. 2025 Jan 25;15:3156. doi: 10.1038/s41598-025-86887-y (PMC11759711; doi:10.1038/s41598-025-86887-y)
Supplement: Supplementary file 2 — Supplementary Information 2. [file 41598_2025_86887_MOESM2_ESM.docx]

**Supplementary Figure S1.** PCA plot based on the frequent (>= 40 individuals) modern taurine breeds present in Run 9 of the 1000 Bull Genomes Project. The 16 ancient cattle and the three parchment samples are projected onto the principal components that represent the genetic variation defined by the modern breeds. The color and shape of the smaller points indicate the modern breed of the individuals. The geographic origin and subspecies of the ancient and parchment individuals is indicated by the color and orientation of the triangles.
