# Supplementary material for: Genomic analysis of three medieval parchments from German monasteries
Source: Sci Rep. 2025 Jan 25;15:3156. doi: 10.1038/s41598-025-86887-y (PMC11759711; doi:10.1038/s41598-025-86887-y)
Supplement: Supplementary file 3 — Supplementary Information 3. [file 41598_2025_86887_MOESM3_ESM.docx]

Supplementary Note: Information about the charters analyzed in our study

**Göttingen, Diplomatic Apparatus of the Georg-August-University / Apparatus diplomaticus, Main Charter Collection App. dipl. 28**

**Digital Copy:**

[DA 28](https://www.monasterium.net/mom/DE-GAUnivGoet/AppDipl/App_dipl_26_27_28/charter)

**Seal:**

Impressed wax seal. The seal image is identical in all three versions (similar but not identical to Heffner, 1875, Plate III No. 30).

**Material:**

Parchment

**Dimensions:**

68 cm height x 53.8 cm width

**Sample Taken in 2019:**

Narrow strip at the bottom left (a prior sample was taken by Dr. Susanne Hummel (Department of Historical Anthropology and Human Ecology, University of Göttingen): 3 round circles, no results)

**Recipient Copy:**

Forgery, late 1180s

**Date:**

February 7, 1131: 7^th^ day before the Ides of February, indiction IX, year of the Lord’s incarnation 1131, during the reign of Lothar, third King of the Romans, 7^th^ year of his reign.

**Place:**

Goslar, Palace

**Alleged Issuer:**

King Lothar III (King since September 13, 1125; Emperor since June 4, 1133; died December 1137)

**Recipient:**

Gerhard, Provost of the Augustinian Canons of Riechenberg and bailiff Hugoldus

**Content:**

King Lothar III of the Romans confirms at the request of Provost Gerhard of Riechenberg and his bailiff Hugoldus that he has taken the church dedicated to the Virgin Mary in Riechenberg under his protection and confirms the donations made by subdeacon and canon Peter of St. Simon and Judas in Goslar on the day of the church’s consecration with the consent of his mother to the church, namely the villa Beningerod, bordered by the swamp near the villa Botingerode and the military road leading from Goslar to Hildesheim, as well as 5 manses in Stapelen and 1 ½ manses in Gerstedt. [Version A:] He further confirms that the church additionally owns properties in the following locations: Beniggerod, Botigerod, Stapelen, Huneriggerod, Hanenthorp, Goslar, Gerstide, Bredenheim, Thorniten, Thornitehusen, Emeleriggerod, Naun, Laggenize, Badenhusen, Hagehusen, Waldenhusen, Pockenhusen, Osdagheshusen, Bukeneim, Bultheim, Maledem, Silegem, Diureshusen, Ebbigerod, Kantigerod, Selede, Herre, Walmoden, a mill in Sledem by the river Ovekare, Biwende, Nienthorp, Puggenstide, Machteresheim, Beddinge, Lieverikestorp, Gravestorp, and the Nordberg bordered by the brooks Scobike and Grana, which the Riechenberg church acquired partly from the Goslar church and partly from others through purchase or exchange. [Version C:] If minerals of any kind are found on the church’s properties, they shall belong to the church. [Version A:] The king further confirms that he affirms all properties the Riechenberg church currently possesses of will acquire in the future and takes them under his protection. 21 witnesses.

**Comment:**

Alleged original in three versions that do no entirely match (cf. Goetting, 1970, pp. 132-166). The three versions are forgeries from different periods: Forgery A = Dipl. App. 28 = DLoIII. 32: Late 1180s, cf Goetting, 1970, pp. 145-154; Böhmer/Petke No. +260. Forgery B = Dipl. App. 26 = DLoIII. 128A: Late 1280s, cf. Goetting, 1970, pp. 154-156; Böhmer/Petke No. +261. Forgery C = Dipl. App. 27 = DLoIII. 128A1: Early 14^th^ century, cf. Goettingen, 1970, pp. 156-158; Böhmer/Petke No. +262.

**Bibiliography**

**Editions:**

- *Urkundenbuch der Stadt Goslar und der in und bei Goslar gelegenen geistlichen Stiftungen*, edited by G. Bode. Vol. 1. Halle 1893 (Geschichtsquellen der Provinz Sachsen; 29), p. 209 No. 177.
- *MGH DLoIII* = Die Urkunden Lothars III. und der Kaiserin Richenza, hg. v. Emil von Ottenthal u. Hans Hirsch. Berlin 1927 (MGH Die Urkunden der deutschen Könige und Kaiser; 8).

**References:**

- Goetting, H. Die Riechenberger Fälschungen und das zweite Königssiegel Lothars III, *Mitteilungen des Instituts für Österreichische Geschichtsforschung* 78, 1970, pp. 132-166.
- Böhmer/Petke = Böhmer, J. F. Regesta Imperii IV: Erste Abteilung: Die Regesten des Kaiserreiches unter Lothar III. und Konrad III. Erster Teil: Lothar III. 1125 (1075-1137), neubearb. v. Wolfgang Petke. Köln-Weimar-Wien 1994, Nr. +260, +261, +262.
- Heffner, C. *Die deutschen Kaiser- und Königs-Siegel nebst denen der Kaiserinnen, Königinnen und Reichsverweser*, Würzbug 1875.

**Göttingen, Diplomatic Apparatus of the Georg-August-University / Apparatus diplomaticus, Main Charter Collection App. dipl. 40**

**Digital Copy:**

[DA 40](https://www.monasterium.net/mom/DE-GAUnivGoet/AppDipl/App_dipl_40/charter)

**Seal:**

Embossed wax seal comes through, attached with a double parchment strip (highly unusual!). The seal image and legend are no longer recognizable.

**Material:**

Parchment with various missing parts due to improper storage

**Dimensions:**

41.3 cm height x 44.6 cm width

**Sample Taken in 2019:**

Bottom right corner next to the seal

**Recipient Copy:**

Forgery, mid or second half of the 12^th^ century

**Date:**

March 26, 1103: year of the Lord’s incarnation 1103, indiction XI, during the reign of King Henry IV, acted in Paderborn on the Lord’s Supper, 7^th^ day before the Kalends of April.

**Place:**

Paderborn, during the synod

**Alleged Issuer:**

Bishop Henry of Paderborn (in office 1084-1127)

**Recipient:**

Benedictine Abbey of Abdinghof, Paderborn

**Content:**

Bishop Henry of Paderborn confirms to the monastery of SS. Peter and Paul in the suburb of Paderborn (Abdinghof) under Abbort Gumpert the following donations made by him at various times: a manse in Curbike (May 3, 1090), a tithe in Liesmari, a serf named Meinhard and his wife Gesike (------) exchanged for a woman named Skicike (?) and her son (---), a fishpond near Hildesheim in Rimbike, a manse in the town previously held in fief by the ministerial Eizo who entered the monastery, a manse in Balhornon previously held in fief by the ministerial Hugo de Gladebach, a manse in Hatheberninchuson, two estates in Bedincthorp and Erpincthorp for a silver jug of 30 marks. 52 witnesses.

**Comment:**

According to Wilmans, 1876, an original from the 12^th^ century. According to Honselmann, 1950, a forgery, recognizable by the dictation: the similar structure, the use of formulas and datings atypical for the time, irregular formulations in the protocol and eschatocol as well as in the arenga; typical script of the Abdinghof school (see also Vita Meinwerci). The format of the parchment is much too large in relation to the insignificance of the content.

**Bibliography**

**Edition:**

- *Westfälisches Urkundenbuch. Additamenta*, edited by Roger Wilmans. Münster 1877, p. 27 No. 25.

**References:**

- Wilmans, R. Die Urkundenfälschungen des Klosters Abdinghof und Vita Meinwerci. *Zeitschrift für vaterländische Geschichte und Altertumskunde (Westfalen)*. 34, 1876, pp. 3-36.
- Honselmann, K. Die sogenannten Abdinghofer Fälschungen. Echte Traditionsnotizen in der Aufmachung von Siegelurkunden. *Westfälische Zeitschrift*. 100, 1950, pp. 292-356

**Göttingen, Diplomatic Apparatus of the Georg-August-University / Apparatus diplomaticus, Main Charter Collection App. dipl. 45**

**Digital Copy:**

[DA 45](https://www.monasterium.net/mom/DE-GAUnivGoet/AppDipl/App_dipl_45/charter)

**Seal:**

Embossed wax seal comes through. Seal image: The bishop seated on his throne in pontifical vestments, holding a crosier in his right hand and an open Gospel book in his left hand. Legend: *BERNHARDVS D*[E]*I GRA*[TIA] *HILDESHEIMENSIS EP*[ISCOPV]*S*.

**Material:**

Parchment

**Dimensions:**

46.9 cm height x 33.5 cm width

**Sample Taken in 2019:**

Small corner piece from the bottom left

**Date:**

November 5, 1133: year of the Lord’s incarnation 1133, indiction XI, 11^th^ day before the Nones of Novembre.

**Place:**

No specification

**Recipient Copy:**

Scribe Riechenberg A

Authentic Document

**Issuer:**

Bishop Bernhard of Hildesheim (in office 1130-1153)

**Recipient:**

Augustinian Canons of Riechenberg near Goslar (Lower-Saxony)

**Content:**

Bishop Bernhard of Hildesheim acknowledges that he consecrated the church built by Provost Gerhard of Riechenberg with his permission in Hanenthorp. He declares the inhabitants of Hanenthorp exempt from the jurisdiction claimed by Arnoldus of Thornthunen and his brother Liudgerus under the church of Thornthune, stating they are parish members of the church of Heriggen. He grants them the baptismal and burial rights in the new church in Hanenthorp and compensates the church of Heriggen with half a manse in Hanenthorp, to which archpriest Bruno, to whose district the church of Heriggen belongs, agrees. 24 named and additional unnamed witnesses.

**Bibliography**

**Edition:**

- *Urkundenbuch der Stadt Goslar und der in und bei Goslar gelegenen geistlichen Stiftungen*, edited by G. Bode. Vol. 1. Halle 1893 (Geschichtsquellen der Provinz Sachsen; 29), p. 209 No. 184.

**Reference:**

- Lücht, W. Neue Beobachtungen zum Güterstreit der Augustiner-Chorherrenstifte Riechenberg und Georgenberg bei Goslar in Verbindung mit der Datierung von D F I 80. *Archiv für Diplomatik, Schriftgeschichte, Siegel- und Wappenkunde.* 60, 2014, pp. 117-134
